# Real world comparison of adjuvant vs. salvage radiation therapy on cancer-control outcomes after radical prostatectomy

**DOI:** 10.1007/s00066-025-02400-4

**Published:** 2025-05-06

**Authors:** Mike Wenzel, Katrin Burdenski, Nikolaos Tselis, Claus Rödel, Christian Brandts, Marit Ahrens, Jens Koellermann, Markus Graefen, Clara Humke, Carolin Siech, Benedikt Hoeh, Severine Banek, Felix K. H. Chun, Philipp Mandel

**Affiliations:** 1 Department of Urology, University Hospital Frankfurt, Goethe University Frankfurt am Main, Frankfurt, Germany; 2 University Cancer Center Frankfurt (UCT), Frankfurt am Main, Germany; 3 Department of Radiation Oncology, University Hospital Frankfurt, Goethe University Frankfurt am Main, Frankfurt, Germany; 4 Department of Hematology/Oncology, University Hospital Frankfurt, Goethe University Frankfurt am Main, Frankfurt, Germany; 5 Department of Pathology, University Hospital Frankfurt, Goethe University Frankfurt am Main, Frankfurt, Germany; 6 Martini-Klinik Prostate Cancer Center, University Hospital Hamburg-Eppendorf, Hamburg, Germany; 7 Department of Urology, Goethe University Hospital Frankfurt, Frankfurt/Main, Germany

**Keywords:** Survival, EBRT, RARP, Radiotherapy, Robotic

## Abstract

**Purpose:**

Outcomes of adjuvant (aRT) or salvage radiation therapy (sRT) after radical prostatectomy are under investigation regarding cancer-control outcomes.

**Methods:**

Relying on the University Cancer Center database elaborating differences in metastasis-free (MFS), cancer-specific (CSS) and overall survival (OS) of aRT vs. sRT-treated patients between 2014–2024. Sensitivity analyses addressed high-risk patients with pN1 and/or Gleason score 8–10 and/or pT3–4 stage.

**Results:**

Of 1862 patients, 7.1% underwent aRT and 93% were in the sRT group. Median PSA at sRT was 0.33 ng/ml. Patients with aRT harbored significantly worse baseline tumor and pathological characteristics such as PSA level (12.0 vs. 7.6 ng/ml), Gleason score 9–10 (30% vs. 9.8%), D’Amico high risk prostate cancer (97% vs. 56%), as well as pT3–4, pN1 and positive surgical margins rates (all *p* < 0.001). Similar observations were made for high-risk patients. No differences were observed for aRT vs. sRT with 60-month MFS rates of 85.1% vs. 95.4% (hazard ratio [HR] 0.60, *p* = 0.18). 60-months CSS-rates of 96.8% vs. 99.1% and 60-month OS-rates of 91.0% vs. 89.1% respectively (all *p* ≥ 0.15). Neither sensitivity analyses of high-risk patients nor multivariable adjusted Cox regression models revealed significant differences regarding MFS, CSS or OS in aRT vs. SRT comparison (all *p* ≥ 0.05), despite aRT showing qualitatively better OS results.

**Conclusion:**

Within real-world setting, patients undergoing aRT harbor wore tumor characteristics. However, these differences did not translate into significant differences of mid-term oncological outcomes, relative to sRT patients. Similar observations were made within analyses of high-risk patients with pT3–4 and/or Gleason 8–10 and/or pN1 stage, nevertheless aRT showed slightly higher OS rates within this subgroup.

## Introduction

For radical prostatectomy-treated prostate cancer patients, European Urology Association (EAU) guidelines strongly recommend adjuvant radiation therapy (aRT) in selected patient cohorts of pN0 patients with Gleason score 8–10 and pT3‑4 stage ± positive surgical margins after surgery [1]. In general, four prospective randomized trials provided better cancer-control outcomes such as biochemical recurrence-free (BCR) survival or overall survival (OS) in aRT patients and especially those with adverse pathological criteria, compared to observation after radical prostatectomy [2–5]. However, aRT may be associated with increased rates of severe side effects and persistent toxicity after initial surgical prostate cancer treatment^6,7^. To reduce the probability of long-lasting severe toxicity rates, salvage radiation therapy (sRT) is discussed as an alternative treatment option. Three previously published randomized-controlled phase III studies and one pooled meta-analysis compared aRT vs. sRT showed no difference in five-year BCR-free survival or OS rates [6, 8–10]. Despite these findings, several other risk factors for worse cancer-control outcomes have been investigated favoring aRT in pN1 patients or prostate cancer patients with at least one of the following risk factors: Gleason score 8–10 and pT3–4 and/or pN1 disease [11–13]. Moreover, previous findings suggested that sRT is more effective in an early salvage setting of PSA < 0.25 ng/ml [14]. Consequently, optimal selection for patients undergoing aRT or sRT after initial radical prostatectomy is still under scientific investigation.

We addressed this unmet need by relying on the University Cancer Center database of a European tertiary-care university hospital to compare cancer-control outcome rates such as metastasis-free survival (MFS) and OS of contemporary aRT vs. sRT patients. We hypothesized that important differences may exist regarding cancer-control outcomes of aRT vs. sRT prostate cancer patients, especially in those with adverse pathological characteristics.

## Materials and methods

### Study population

Patient data were provided by the University Cancer Center Frankfurt (UCT) after obtaining approval from the local ethics committee (reference number: SUG-4-2024) and adhering to the principles of the Declaration of Helsinki. We conducted a retrospective analysis of all prostate cancer patients treated with radical prostatectomy with a curative intent at the Department of Urology or the Department of Radiation Oncology at University Hospital Frankfurt, Germany, between 2014 and 2024. All treatment indications for prostate cancer were determined by a multidisciplinary tumor board. Patients with metastatic disease at the time of diagnosis or those with unknown primary treatment were excluded. Based on these criteria, 1862 patients with radical prostatectomy treatment and subsequent radiation therapy were included in the current study. Moreover, for further subgroup analyses, 55 patients with BCR and no RT were identified as competitors (total *n* = 1917).

### aRT vs. sRT

Patients received aRT or sRT after initial radical prostatectomy, based on pathological characteristics and patients’ preferences after case discussion within a multidisciplinary tumor board. aRT was defined as radiation therapy to at least the prostatic bed within six months from initial radical prostatectomy and undetectable PSA (*n* = 132). Patients in the sRT group either underwent RT in case of BCR (*n* = 210) or did not develop BCR (*n* = 1520). Patients with BCR and still not undergoing RT were grouped as an additional no RT control group (*n* = 55). aRT and sRT were delivered as external beam radiation therapy (EBRT) in accordance with the respective guideline recommendations at time of treatment.

### Statistical analysis

Descriptive statistics involved calculating the frequencies and proportions of the categorical variables included in the analysis. For continuous variables, median values and interquartile ranges (IQR) were reported. The Chi-square test was used to evaluate the statistical significance of differences in proportions, while the t‑test and Kruskal-Wallis test were applied to analyze differences in distributions.

In the first step of analyses, MFS and OS outcomes were depicted in Kaplan-Meier curve analyses for all aRT vs. sRT patients after initial radical prostatectomy. Subsequently, sensitivity analyses addressed patients with adverse characteristics such as Gleason score 8–10 and/or pT3–4 stage and/or pN1 lymph node disease (“high-risk”). Univariable as well as multivariable Cox regression models were computed for all analyses. Multivariable adjustment was performed in order to maximally adjust for potential confounders of baseline patient (age, Eastern Cooperative Oncology Group [ECOG] status) or tumor characteristics (cT stage, cN1 stage, Gleason score, positive surgical margins, use of ADT) between aRT vs. sRT patients. All tests were two-sided with a level of significance set at *p* < 0.05. R software environment for statistical computing and graphics (version 3.4.3) was used for all analyses.

## Results

Of 1862 patients with initial radical prostatectomy qualified for analyses, 132 (7.1%) underwent aRT vs. 1730 (93%) grouped in the sRT cohort, of which 12% received a radiotherapy at time of censoring. Overall, patients had median age of 66 years and an initial PSA level of 7.8 ng/ml. Most patients harbored ECOG status 0 (94%) and harbored D’Amico high risk prostate cancer (59%). Median follow-up was 19 months (IQR: 1–40 months).

### Baseline characteristics: aRT vs. sRT

In comparison between aRT vs. sRT patients (Table 1), patients with aRT harbored higher initial PSA level (12.0 vs. 7.6 ng/ml) and higher number of positive cores (8 vs. 5), as well as percentage of positive cores at biopsy (75% vs. 60%, all *p* < 0.001). Moreover, patients with aRT harbored more frequently PSA levels ≥ 20 ng/nl (28% vs. 11%), Gleason score 9–10 (30% vs. 9.8%) and high-risk prostate cancer (97% vs. 56%, all *p* < 0.001). Similarly, rates of cT3–4, pT3–4, cN1, pN1 and positive surgical margins were significantly higher for aRT patients, relative to sRT patients (all *p* < 0.001). Median PSA at sRT was 0.33 ng/ml (IQR: 0.20–0.61).

**Table 1:** Descriptive characteristics of 1862 prostate cancer patients treated with adjuvant radiation therapy (aRT) vs. salvage radiation therapy (sRT) after radical prostatectomy

Characteristic	*N*	Overall *N* = 1,862^1^	aRT, *N* = 132 (7.1%)^1^	sRT, *N* = 1730 (93%)^1^	*p*-value^2^
Age, years	1862	66 (60, 71)	65 (60, 70)	66 (61, 71)	0.077
PSA, ng/ml	1787	7.8 (5.4, 12.0)	12.0 (7.6, 22.3)	7.6 (5.3, 11.3)	< 0.001
BMI	166	26.2 (24.4, 28.7)	26.9 (24.8, 29.7)	26.0 (24.2, 27.7)	0.14
Positive cores at biopsy	1469	5 (3, 7)	8 (5, 12)	5 (3, 7)	< 0.001
Percentage of positive cores at biopsy	1261	60 (30, 80)	75 (50, 100)	60 (30, 80)	< 0.001
*ECOG status*	1738	–			0.7
0		1632 (94%)	108 (95%)	1524 (94%)	
1–2		106 (6.1%)	6 (5.3%)	100 (6.2%)	
PSA category	1787	–			< 0.001
< 10 ng/ml		1187 (66%)	46 (39%)	1141 (68%)	
10–20 ng/ml		382 (21%)	40 (34%)	342 (21%)	
≥ 20 ng/ml		218 (12%)	33 (28%)	185 (11%)	
*Gleason Score*	1855	–			< 0.001
6		369 (20%)	0 (0%)	369 (21%)	
7a		694 (37%)	26 (20%)	668 (39%)	
7b		365 (20%)	45 (34%)	320 (19%)	
8		219 (12%)	22 (17%)	197 (11%)	
9–10		208 (11%)	39 (30%)	169 (9.8%)	
*D’Amico risk group*	1860	–			< 0.001
Low		203 (11%)	0 (0%)	203 (12%)	
Intermediate		568 (31%)	4 (3.0%)	564 (33%)	
High		1089 (59%)	128 (97%)	961 (56%)	
*cT stage*	1848	–			< 0.001
cT1–2		983 (53%)	11 (8.3%)	972 (57%)	
cT3–4		865 (47%)	121 (92%)	744 (43%)	
*pT stage*	1853	–			< 0.001
pT1–2		979 (53%)	11 (8.3%)	968 (57%)	
pT3–4		863 (47%)	121 (92%)	742 (43%)	
*cN stage*	1861	–			< 0.001
cN0		1681 (90%)	70 (53%)	1611 (93%)	
cN1		180 (9.7%)	62 (47%)	118 (6.8%)	
*pN stage*	1862	–			< 0.001
pN0		1460 (78%)	70 (53%)	1390 (80%)	
pN1		177 (9.5%)	60 (45%)	117 (6.8%)	
pNx		225 (12%)	2 (1.5%)	223 (13%)	
*Surgical margin*	1862	–			< 0.001
R0		1253 (67%)	32 (24%)	1221 (71%)	
R1		552 (30%)	97 (73%)	455 (26%)	
Rx		57 (3.1%)	3 (2.3%)	54 (3.1%)	

*PSA* Prostate-specific antigen, *BMI* Body-mass index, *ECOG* Eastern Cooperative Oncology Group

^1^ Median (Q1, Q3); *n* (%)

^2^ Kruskal-Wallis rank sum test; Fisher’s exact test; Pearson’s Chi-square test

### Baseline characteristics of high-risk patients: aRT vs. sRT

In comparison of 994 patients with adverse pathological characteristics (pT3–4 and/or Gleason 8–10 and/or pN1 stage), 127 (13%) underwent aRT vs. 867 (87%) sRT (Table 2). Here, patients with sRT were significantly older than aRT patients (67 vs. 65, years, *p* < 0.01). Moreover, similarly, as in the overall cohort, patients in the aRT cohort harbored significantly more frequently unfavorable tumor characteristics such as positive cores, PSA ≥ 20 ng/ml, Gleason 8–10, cT3–4 stage, pT3–4 stage, cN1 stage, pN1 stage or positive surgical margins. Median PSA in this sRT cohort was 0.38 ng/ml (IQR: 0.22–0.69).

**Table 2:** Descriptive characteristics of 994 prostate cancer patients treated with adjuvant radiation therapy (aRT) vs. salvage radiation therapy (sRT) after radical prostatectomy and high risk pathological features (Gleason Score 8‑10 and/or pT3‑4. And/or pN1)

Characteristic	*N*	Overall N = 994^1^	aRT, *N* = 127 (13%)^1^	sRT, *N* = 867 (87%)^1^	*p*-value^2^
Age, years	994	67 (62, 72)	65 (59, 70)	67 (62, 72)	0.002
PSA, ng/ml	949	9 (6, 16)	12 (8, 23)	9 (6, 15)	< 0.001
BMI	114	26.5 (24.7, 28.7)	26.9 (24.8, 29.7)	26.4 (24.5, 28.4)	0.3
Positive cores at biopsy	763	6 (3, 9)	9 (5, 12)	6 (3, 8)	< 0.001
Percentage of positive cores at biopsy	659	70 (40, 90)	75 (50, 100)	70 (40, 90)	0.13
*ECOG status*	928	–			0.4
0		858 (92%)	104 (95%)	754 (92%)	
1–2		70 (7.5%)	6 (5.5%)	64 (7.8%)	
*PSA category*	949	–			< 0.001
< 10 ng/ml		523 (55%)	44 (39%)	479 (57%)	
10–20 ng/ml		236 (25%)	37 (32%)	199 (24%)	
≥ 20 ng/ml		190 (20%)	33 (29%)	157 (19%)	
*Gleason Score*	992	–			< 0.001
6		81 (8.2%)	0 (0%)	81 (9.4%)	
7a		261 (26%)	22 (17%)	239 (28%)	
7b		223 (22%)	44 (35%)	179 (21%)	
8		219 (22%)	22 (17%)	197 (23%)	
9–10		208 (21%)	39 (31%)	169 (20%)	
*D’Amico risk group*	993	–			0.6
Low		0 (0%)	0 (0%)	0 (0%)	
Intermediate		11 (1.1%)	2 (1.6%)	9 (1.0%)	
High		982 (99%)	125 (98%)	857 (99%)	
*cT stage*	991	–			0.004
cT1–2		126 (13%)	6 (4.7%)	120 (14%)	
cT3–4		865 (87%)	121 (95%)	744 (86%)	
*pT stage*	988	–			0.004
pT1–2		125 (13%)	6 (4.7%)	119 (14%)	
pT3–4		863 (87%)	121 (95%)	742 (86%)	
*cN stage*	994	–			< 0.001
cN0		814 (82%)	65 (51%)	749 (86%)	
cN1		180 (18%)	62 (49%)	118 (14%)	
*pN stage*	994	–			< 0.001
pN0		757 (76%)	65 (51%)	692 (80%)	
pN1		177 (18%)	60 (47%)	117 (13%)	
pNx		60 (6.0%)	2 (1.6%)	58 (6.7%)	
*Surgical margin*	994	–			< 0.001
R0		501 (50%)	29 (23%)	472 (54%)	
R1		454 (46%)	95 (75%)	359 (41%)	
Rx		39 (3.9%)	3 (2.4%)	36 (4.2%)	

*PSA* Prostate-specific antigen, *BMI* Body-mass index, *ECOG* Eastern Cooperative Oncology Group

^1^ Median (Q1, Q3); *n* (%)

^2^ Kruskal-Wallis rank sum test; Fisher’s exact test; Pearson’s Chi-square test

### MFS in aRT vs. sRT (vs. no RT) patients

**Fig. 1:**
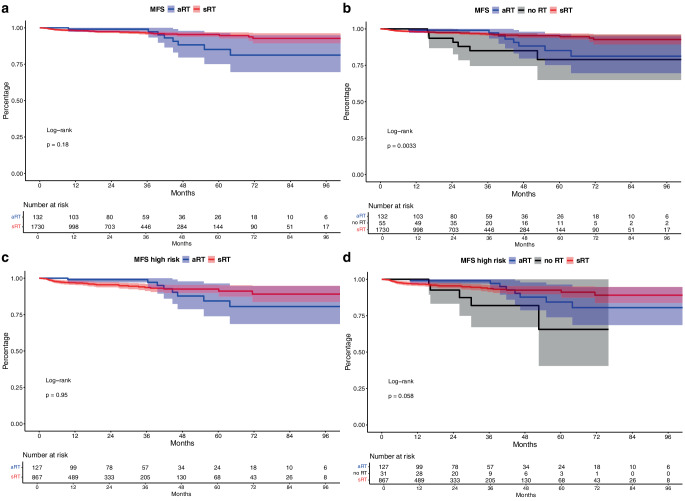
Kaplan-Meier curves depicting metastasis-free survival (MFS) of patients receiving adjuvant radiation therapy (aRT) vs. salvage radiation therapy (sRT, **a**) vs. no radiation therapy (**b**). Subgroup analyses focused on high risk patients with adverse pathological characteristics (**c**,**d**)

Regarding MFS analyses, no significant differences between aRT vs. sRT patients were observed (Fig. 1a, *p* = 0.18), with a corresponding hazard ratio (HR) of 0.60 and 60-months MFS rates of 85.1% vs. 95.4%. Incorporating patients receiving no RT (Fig. 1b), showed a significant difference between all three groups (*p* < 0.01), with a 60-month MFS rate for no RT of 75.0% (HR relative to aRT: 2.1, *p* = 0.15). After controlling for patient and tumor characteristics in multivariable Cox regression models, no difference between all three groups remained with a trend towards higher rate of metastasis in no RT patients, relative to aRT (HR: 3.68, *p* = 0.08).

Sensitivity analyses of patients with high-risk pathological features (Fig. 1c, d), showed 60-month MFS rates of 84.4% vs. 92.5% vs. 65.6% for aRT vs. sRT vs. no RT. In multivariable Cox regression models, no difference between aRT vs. sRT was observed (HR: 1.90, *p* = 0.2).

### CSS in aRT vs. sRT (vs. no RT) patients

In CSS analyses, no significant differences between aRT vs. SRT (vs. no RT) were observed (Fig. 1a, b, both *p* ≥ 0.4) with 60 months CSS rates of 96.8% vs. 99.1% vs. 98.1% for aRT vs. sRT vs. no RT. In multivariable Cox regression models adjusting for unfavorable patient and tumor characteristics, no differences between aRT vs. sRT was observed (*p* = 0.2) with a trend towards more unfavorable CSS outcomes for no RT patients (HR: 13.9, *p* = 0.09).

**Fig. 2:**
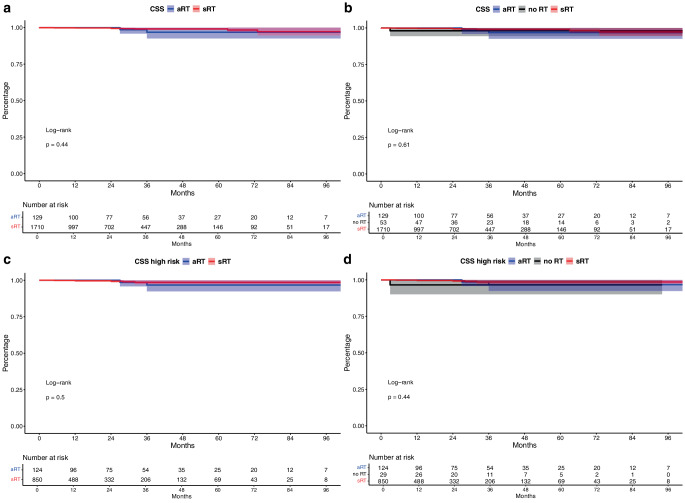
Kaplan-Meier curves depicting cancer-specific survival (CSS) of patients receiving adjuvant radiation therapy (aRT) vs. salvage radiation therapy (sRT, **a**) vs. no radiation therapy (**b**). Subgroup analyses focused on high-risk patients with adverse pathological characteristics (**c**,**d**)

In subsequent CSS analyses of high-risk patients with pT3–4 and/or Gleason 8–10 and/or pN1 stage, also no differences were observed (both *p* ≥ 0.4, Fig. 2c, d), with 60-months CSS of 96.7% vs. 98.6% vs. 96.6% for aRT vs. sRT vs. no RT. In adjusted multivariable Cox regression models, no difference between aRT and sRT was observed (*p* = 0.2) with a significant higher risk of cancer-specific mortality for no RT patients (HR: 25.5, *p* = 0.037).

### OS in aRT vs. sRT (vs. no RT) patients

**Fig. 3:**
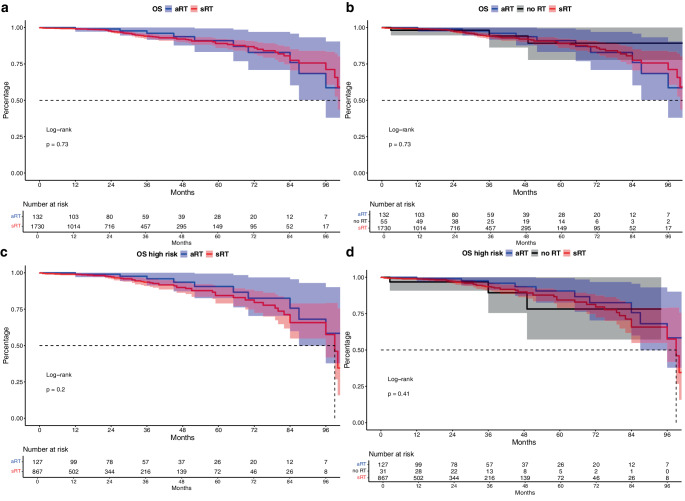
Kaplan-Meier curves depicting overall survival (OS) of patients receiving adjuvant radiation therapy (aRT) vs. salvage radiation therapy (sRT, **a**) vs. no radiation therapy (**b**). Subgroup analyses focused on high risk patients with adverse pathological characteristics (**c**,**d**)

In final OS analyses, also no statistically significant differences between aRT vs. sRT (vs. no RT) patients were observed (Fig. 3a, b, both *p* = 0.7), with 60-months OS of 91.0% vs. 89.1% vs. 89.3% for aRT vs. sRT vs. no RT. After multivariable adjustment, neither sRT nor no RT were associated with higher all-cause mortality risk, relative to aRT patients (both *p* ≥ 0.15).

In high-risk patients (Fig. 3c, d), 60 months OS rates were 90.6% vs. 84.4% vs. 78.2% for aRT vs. sRT vs. no RT. These results were not statistically significant but aRT showed qualitatively better OS rates in this subgroup (all *p* ≥ 0.1).

## Discussion

We hypothesized that important differences regarding cancer-control outcomes of aRT vs. sRT prostate cancer patients may exist. Moreover, we also hypothesized that this effect may be especially present in those patients with adverse pathological characteristics after radical prostatectomy. To test for these assumptions, we relied on the University Cancer Center database of a European tertiary-care hospital and made several noteworthy observations.

First, we observed that differences in tumor characteristics of prostate cancer patients undergoing aRT vs. sRT exist. More specifically, patients undergoing aRT harbored higher PSA level prior to radical prostatectomy (12.0 vs. 7.6 ng/ml), higher tumor burden, approximated by the number of positive cores and percentage of positive cores, as well as proportions of Gleason score 9–10, D’Amico high-risk rates, as well as rates of pT3–4, cN1, pN1 and positive surgical margins. In subgroup analyses of patients with adverse pathological features after radical prostatectomy only (pT3–4 and/or Gleason 8–10 and/or pN1 disease), virtually similar observations regarding higher rates of Gleason 8–10, pT3–4 stage, pN1 stage or positive surgical margins within the aRT vs. sRT group were observed. Moreover, sRT were also significantly older than aRT patients. Comparing these findings to previously published cohorts, the findings of our study are consistent. For example, in a report by Tilki et al., comparing aRT vs. sRT within a multi-center study, aRT also harbored significantly worse baseline prostate cancer, as well as pathological characteristics, such as higher PSA, cT stage, rates of positive surgical margins, or pN1 stage [11]. Moreover, in an additional subgroup analysis, within this study, the authors also replicated these findings in patients with adverse pathologies. These findings are not surprising since guidelines explicitly recommend aRT in patients with adverse pathological characteristics, which may be estimated by initial tumor characteristics such as stage, grade and PSA level prior to radical prostatectomy [1, 15–17]. Moreover, older age in sRT with unfavorable pathological characteristics is also not surprising, since in more elderly patients aRT may be recommended less frequently as elderly patients do have a shorter life expectancy and to avoid possibly side effect, which may be more frequently observed in elderly patients [7].

Second, we also made important observations regarding frequency of applied aRTs. Specifically, within the overall cohort, 7.1% of all patients received aRT and within the subgroup of prostate cancer patients with adverse postsurgical pathological features only 13% underwent aRT. These numbers are lower than previously reported numbers by the above discussed multi-center study by Tilki et al. of patients sampled between 1989 and 2016 in which 15% of the overall cohort and 42% of patients with adverse pathology underwent aRT [11]. Similarly, a report by Hwang et al. in which patients with ≥ pT3 disease or positive surgical margins are sampled between 1987 and 2013, an aRT rate of 23.7% was recorded [18]. However, lower numbers within our cohort may be explained by three reasons. First, the inclusion period of the current study is more recent with years of 2014 to 2024. Compared to the above discussed more historical sampling reports, guideline recommendations may have changed and therefore fewer patients may have received aRT and underwent sRT. Second, the current report may underestimate rates of aRT since not all patients who underwent radical prostatectomy at our hospital necessarily also underwent aRT at our institution. Finally, a recent study by Sigg et al. showed that 44% of all patients refused aRT after multidisciplinary case discussion and recommendation of aRT due to fear of radiation damage [19]. Moreover, it is also of note that within our study cohort of sRT patients, only 12% received post-operative RT due to BCR event, which is only slightly higher than the aRT rate. However, compared to the pooled meta-analysis of the three available prospective trials, at time of censoring, 39% received a sRT [10]. With longer follow-up, BCR and following sRT may also increase in our cohort. These rates indicate that a high number of patients may have received overtreatment, if everyone would have undergone aRT.

Finally, when cancer-control outcomes were compared between aRT vs. sRT patients, also important observations were made. In MFS outcome analyses, no differences between aRT vs. sRT patients were observed, nether in subgroup analyses of patients with adverse pathological features. When patients were incorporated who underwent no RT when BCR occurs, significantly lower MFS rate was observed in patients undergoing aRT or sRT. Similarly, these observations were also seen within the high-risk cohort, also failing statistical significance, probably due to low number of patients included (*n* = 31, *p* = 0.058). Moreover, in CSS and OS analyses, no statistically significant differences between aRT vs. sRT patients were observed, neither in the entire cohort nor in the subgroup analyses of high-risk patients with adverse pathological features. However, aRT was numerical better in the patient cohort with adverse pathological features. Finally, in multivariable Cox regression models, adjusting for unfavorable baseline tumor, pathological and surgical characteristics, also no difference between aRT vs. sRT was observed. One possible reason for the insignificant results might be the fact, that no information regarding the PSA at time of sRT was available and our cohort might be underpowered. Our findings are consistent with findings from Tilki et al. showing no differences in OS in all patients regarding aRT vs. sRT [11]. Nonetheless patients with sRT harbored better OS prognosis than without RT. Moreover, Tilki et al. could show that treatment at an earlier PSA level is associated with better outcomes. Within our study, median PSA level at sRT was 0.33 ng/ml, which was only slightly higher than the suggested best threshold of ≤ 0.25 ng/ml. Moreover, Tilki et al. provided an OS benefit in patients with adverse pathology, which could only be proved qualitatively in our study, maybe due to differences in number of events and median follow up, which were both substantially lower within our study. Similar to our findings, the three currently available prospective randomized controlled trials also found no difference in disease relapsing within short term follow up duration [6, 8–10]. However, one needs to keep in mind that some of these trials terminated due to insufficient recruitment despite other limitations. Also similar to our findings, a recently published multicenter cohort of 510 pT3 patients showed no difference between MFS and OS rates in comparison between aRT vs. sRT [20]. Conversely, other studies suggest better outcomes in aRT patients regarding cancer-control outcomes, especially when adverse pathological features were present [18, 21–23]. However, most of these studies relied on a more historical patient sample. Nonetheless it needs to be emphasized that the above discussed prospective studies, as well as retrospective studies finding no difference in aRT vs. sRT patients, are limited due to the inclusion of a high number of patients with low to intermediate risk prostate cancer which may never harbor a BCR but underwent aRT. This limitation is also shared by the current study. However, to overcome this limitation, our subgroup analyses focused exclusively on almost 1000 patients with adverse pathological characteristics [24].

Despite the discussed limitations of the current study, further points should be acknowledged in its interpretation such as the retrospective, non-randomized single-center design. Unfortunately, no information regarding length and influence of androgen deprivation therapy or the exact extent of the RT technique, target volume and dose to the pelvis (±pelvic lymph nodes) were available. Moreover, with better staging modalities and advanced usage of PSMA-PET/CT, better sRT outcomes may be expected in the recent years due to better radiation field selection [25]. For some small subgroups not all information were available and heterogeneous inclusion criteria may differed between some groups. Finally, sRT rates may increase with longer follow-up duration and may influence outcomes.

Taken together, the current study provided robust information regarding patient and tumor characteristics of aRT vs. sRT patients. Specifically, aRT harbor worse characteristics when compared to sRT patients, even in subgroup analyses of patients with adverse pathological features such as pT3–4 and/or Gleason 8–10 and/or pN1 disease. Moreover, current rates of aRT are lower than previously reported data. However, within our study, no statistically significant differences in cancer-control outcomes could be observed between aRT vs. sRT, nevertheless aRT seems to be numerical better especially in patients with adverse pathology. Still, if more patients undergo aRT, the risk of an overtreatment also increases.
